# Differentials in Contraceptive Use Among Selected Minority Ethnic Groups in Nigeria

**DOI:** 10.3389/fgwh.2022.878779

**Published:** 2022-06-03

**Authors:** Aboluwaji Daniel Ayinmoro, Olufunke A. Fayehun

**Affiliations:** Department of Sociology, Faculty of the Social Sciences, University of Ibadan, Ibadan, Nigeria

**Keywords:** modern contraceptive use, husbands' disapproval, side effects, minority ethnic groups, Nigeria

## Abstract

Ethnicity is one of the critical factors that shape contraceptive use in Nigeria. While there are growing disparities in contraceptive uptake among women of reproductive age in the three major ethnic groups (Hausa, Igbo and Yoruba), not much is known about differentials in contraceptive use among the minority ethnic groups. This study examined differentials in contraceptive use among a sample of 1,072 respondents comprising the Ebira (352), Igala (358) and Okun (361) ethnic groups in Nigeria. Questionnaire was administered to respondents proportionately in the selected minority ethnic groups with six key informant interviews and 12 focus group discussions to generate quantitative and qualitative data among ever-married women. Quantitative data were analyzed at bivariable and multivariable levels. The qualitative data were content-analyzed. Differentials in contraception are shaped by ethnic affiliations and socio-demographic characteristics of couples. The use of modern contraceptives was low among the Ebira (25.7%) and Igala (24.1%) ethnic groups, but high among Okun (67%) women of reproductive age. The odd of using a modern contraceptive is significantly higher among the Okun women (UOR = 5.618, 95% CI 4.068–7.759) than the Ebira and Igala. There is no significant difference between the Ebira and Igala minority ethnic groups on modern contraceptive use. Ethnicity as a factor is not a stand-alone predictor of the use of modern contraception among the study groups, other socio-economic variables such as residence, religion, income and marital status were significant predictors of modern contraceptive use among minority ethnic groups. We suggest introducing reproductive health intervention programmes targeted at sensitizing the minority ethnic groups on effective modern contraceptive use while addressing their specific modern contraceptive need in Nigeria.

## Introduction

There has been a global increase in the last two decades in the estimates of women using a modern contraceptive method from 663 million to 851 million and contraceptive prevalence rate (CPR) from 47.7 to 49.0 per cent (1). Despite this increase, there are significant variations in the pattern of contraceptive use between regions and countries of the world, including those at the sub-national levels (2–4). The identified factors for the observed differentials at the macro-level of society are primarily at micro-level of individual and household, including demographic, biological, socio-economic, and behavioral variables (5–10). The cumulative effects of these differentials at micro levels cascade to the macro-level fertility outcomes and uneven uptake of contraception.

Specifically, studies have persistently associated contraceptive use differentials to ethnic affiliations (11–15). Other studies (6, 16–21) posit that ethnicity or race as a factor is not a stand-alone predictor of the use of contraception in both high income and low-income countries. The differentials in contraceptive use are often related to religion, work status, migration status, geographical location and education.

In addition, studies have persistently shown that ethnicity is associated with the method of contraceptive use among women of reproductive age (12, 18, 22–24). For example a study found that less effective contraceptive methods were prominently used among Blacks and Latinas than the White group (12). Other studies also emphasized the ethnic differentials in modern contraceptive use (22, 24).

In sub-Saharan African countries, there has been evidence of modern contraceptive use differentials among ethnic groups (25–30). For instance, a study in Malawi showed that Tonga women are less likely to use modern contraceptives than Nyanga women (29). Also, women from the Diola ethnic group in Senegal are less likely to use a modern contraceptive method than those from the Wolof ethnic group (30). Ethnic contraceptive use differentials were also observed among adolescent girls in Ghana, where adolescents from the Ewe Ga Dagme and Mole-Dagbani ethnic groups were more likely to practice abstinence as contraceptive method than those from the Akan ethnic group (25).

Studies in Nigeria on fertility and contraception have majorly focused on the three major ethnic groups and the geopolitical regions; there is sparse information on minority ethnic groups' contraceptive use (14, 31–34). The 17% contraceptive use prevalence rate (CPR) and 12% CPR reported among currently married women in 2018 NDHS (35) is an aggregation of the minority ethnic groups CPRs in the country. This study, therefore, examines the differences in modern contraceptive use among three selected minority ethnic groups – Ebira, Igala and Okun in Kogi State, North-Central Nigeria.

## Materials and Methods

This study adopted a comparative cross-sectional survey design to elicit data from three minority ethnic groups in Nigeria: Ebira, Igala and Okun of Kogi State. Kogi state is located in the North-central region of Nigeria and has a high population density of minority ethnic groups. For this study, a total sample of 1,072 was estimated using Cochran's (36) sample size determination formula. This sample size was further proportionally distributed among the three selected ethnic groups - Ebira (358), Igala (353) and Okun (361). The Ebira and Igala people are historically traced to the Jukuns of the Kwararafa state, a minority ethnic group in northeast (a kingdom situated in Taraba State) Nigeria characterized by high fertility. On the other hand, the Okun people are traditionally traced to the Yoruba, a major ethnic group in southwest Nigeria characterized by relatively low fertility compared to other geopolitical zones (35).

This study adopted a sequential mixed-method approach to collect data from ever-married women (aged 15–49 years) and ever-married male leaders in the community. The initial information about ethnic membership was provided by the recognized leaders and authorities of hometown associations (HTAs) of the three ethnic groups. After that, respondent-driven sampling was used to administer a structured questionnaire on women in each of the selected ethnic groups based on the estimated sample size for each ethnic group. First sets of respondents were identified in each of the ethnic groups before referrals were made to recruit others until the sample size was completed. Also, six key informant interviews, two from each ethnic group, were purposively conducted with ever-married male leaders, while 12 focus group discussions were held among ever-married women on purpose.

### Outcome and Exposure Variables

The outcome variable is the use of modern contraceptives. Modern contraceptives methods include female sterilization, male sterilization, implants, intrauterine device (IUD), injectables, male condom, female condom, diaphragm, pills, foam or jelly and emergency contraception (37). Modern contraceptive use was re-categorized into a dichotomous variable in this study; those who indicated use as “1” and non-use as “0”. The exposure variables were ethnicity (1 = Ebira, 2 = Igala and 3 = Okun), age (1 = 15–19; 2 = 20–24; 3 = 25–29; 4 = 0–34; 5 = 35–39; 6 = 40–44; 7 = 45–49), residence (1 = rural, 2 = urban), education (1 = no formal education, 2 = primary, 3 = secondary and higher), religion (1 = Catholic, 2 = Other Christians, 3 = Islam, 4 = Traditionalist), occupation (1 = government employees, 2 = private/self-employed, 3 = unemployed), average income (1 = below N30000, 2 = 30,000–49,999, 3 = 50,000 and above), marital status (1 = ever-married, 2 = cohabiting, 3 = divorced/separated) and family type (1 = monogamy, 2 = polygamy).

### Data Analysis

The statistical analysis was at bivariable and multivariable levels for the quantitative data. The qualitative data was analyzed by themes that reflect the study objectives. The bivariate analysis, including frequency counts, cross tab and averages, showed the background characteristics and pattern of modern contraceptive methods used in the study population. The predictive influence of ethnicity and selected variables on modern contraceptive use was explored using a binary logistic at a *p* < 0.05 significance threshold. The analysis was based on the assumption that being a member of a minority ethnic group would exert a certain level of predictive influence on modern contraceptive use as well as interact with other variables. Therefore, the analysis showed adjusted and unadjusted odds on the modern contraceptive use in the study population. The qualitative data was content analyzed based on the themes generated from the interviews and discussion sessions held with the participants.

### Ethical Considerations

Ethical clearance certificates were obtained from the Kogi State Ministry of Health Ethical Review Committee (MOH/KGS/1376/1/95) and the University of Ibadan Social Sciences and Humanities Research Ethics Committee (UI/SSHEC/2018/004). In addition, a written informed consent form was obtained from each respondent while ethical issues on voluntariness, autonomy, confidentiality and cultural sensitivity were considered in the study. The quantitative and qualitative data collection fieldwork occurred between April and July 2018.

## Results

Table 1 presents the background characteristics of study participants by ethnic groups. The average age of participants ranged from 32 to 35 years, with Okun ethnic group having older women within the reproductive age groups than others. Most of the participants across the three minority ethnic groups were married or cohabiting and in monogamous families. While at least three out of five participants resided in urban areas, the Ebira had the highest percentage (75.4%) of urban residents than the Igala (63.2%) and the Okun (60.9%).

**Table 1:** Socio-demographic characteristics of the respondents.

	**Ethnic groups**		
**Demographic Variables**	**Ebira (*n* = 358)**	**Igala (*n* = 353)**	**Okun (*n* = 361)**
**Age**			
15–19	-	-	-
20–24	4.2	1.4	3.9
25–29	31.6	17.6	16.3
30–34	26.5	37.4	28.0
35–39	20.9	25.8	26.9
40–44	10.6	13.6	14.1
45–49	6.1	4.2	10.8
*Mean* (34.4 ± 6.2 years)	32.30 ± 6.26 years	34.04 ± 5.40 years	35.00 ± 6.60 years
**Marital status**			
Ever-married	86.9	95.2	92.2
Cohabiting	7.5	2.0	5.8
Divorced/Separated	5.6	2.8	1.9
**Family type**			
Monogamy	71.2	79.3	87.5
Polygyny	28.8	20.7	12.5
Residence			
Rural	24.6	36.8	39.1
Urban	75.4	63.2	60.9
**Education**			
No formal education	13.7	20.4	14.7
Primary	4.7	12.5	3.9
Secondary and higher	81.6	67.1	81.4
**Occupation**			
Government employees	39.1	44.5	55.1
Private/self-employed	57.5	50.4	40.4
Unemployed	3.4	5.1	4.4
**Average monthly income (N)**			
Below 30,000	81.6	75.4	78.9
30,000–49,999	10.3	15.9	12.2
50,000 and above	8.1	8.8	8.9
**Religion**			
Catholic	7.8	9.1	12.3
Other Christians	45.1	37.4	82.9
Islam	45.9	51.6	3.9
Traditionalist	1.1	2.0	0.8

The proportion of women with formal education is lower among the Igala than the Ebira and Okun as one out of five Igala women had no formal education. However, more Igala women of reproductive age worked in private or self-owned establishments than other minority ethnic groups within the same state. Significantly, few women were unemployed among all the selected ethnic groups. Still, the average monthly income of most of those employed was less than the minimum wage of N30000 (an approximate equivalent of $72). The religious affiliation of women in this study showed some variations among the three ethnic groups. Okun women were predominantly Christians, while women in the other ethnic groups practiced Islam and Christianity in almost equal proportion.

### Modern Contraceptive Use Among Minority Ethnic Groups in Nigeria

The pattern of modern contraceptive use was examined among the selected minority ethnic groups within the same state in North Central Nigeria (Table 2). The prevalence was highest among Okun women of reproductive age (67%); more than twice the proportion of women in the Ebira (25.7%) and Igala (24.1%) minority ethnic groups using the same methods.

**Table 2:** Percentage distribution of respondents currently using modern contraceptive.

**Modern contraceptive methods**	**Ebira (*n* = 358)**	**Igala (*n* = 353)**	**Okun (*n* = 361)**
Any modern method	25.7	24.1	67.0
Male condom	5.9	7.9	29.3
Injectables	8.1	4.2	17.6
Pills	5.6	6.8	17.6
Implants	2.5	1.4	10.9
IUD	2.5	2.8	8.9
Emergency contraception	2.0	1.4	6.1
Female condom	0.6	0.8	5.6
Foam or jelly	0.6	0.6	4.7
Diaphragm	0.6	-	4.5
Female sterilization	0.6	-	1.4
Male sterilization	-	-	0.8

The three most commonly used modern contraceptive methods among the Okun women were male condoms (29.3%), injectables (17.6%) and pills (17.6%). Although these were most frequently used among the other minority ethnic groups, the most modern contraceptives method used among the Igala and Ebira were Injectables and pills, respectively. It is also noteworthy that many Okun (10.9%) women used the implants, unlike other ethnic groups. None of the study participants among the Igala and Ebira reported using male sterilization. Also, diaphragm and female sterilization were not used among Ebira women of reproductive age.

Analysis of the qualitative response on the reasons for the overall low use of modern contraceptives, especially among the Ebira and Igala, showed the subtle effect of beliefs, values and norms in adopting contraceptives. Perceived reasons include the fear of its side effects, husband disapproval because of depletion of sexual pleasure and sin against religious beliefs. Another reason was the preference for traditional methods; the male participants, especially among Ebira and Igala, commonly used traditional methods such as magical rings (oroka), withdrawal and herbs. The perceived efficacy of the traditional methods discourage most couples in the Ebira and Igala ethnic groups from the use of modern contraceptives. When asked whether they used any method of contraception, only a few indicated that they did, while nonusers explained why they did not.

***Female Group*** - P1: Yes, I used injection method, because it's better for me. P2: Yes, I use herbs due to the side effects of modern methods. P3: No, am not using any for now because of the side effects. P4: No, am not using any, because am not yet ready to use. P6: No, am not using any method due to their side effects. P5: I use herbs, because it is more effective.

***Male Group*** - P1: Am not using anyone for now. I have used condom before. I don't enjoy it well. P2: I use condom, but the pleasure was not there. P3: Am not using for now, because of my own belief. P4: I am not using condom but I am using withdrawal method, based on the side effects of the modern methods. P5: I use withdrawal method. P6: I use rings, which I inherited from my father. It worked easily and very effective. Because some condoms may even get torn during intercourse and result to pregnancy that you don't intend to have, but ring (*oroka*) will not fail provided you put it on during intercourse **(Ebira ethnic group)**.

***Female Group*** - P1: It is the one that your body can take that you take. Because some people prefer injectables, while some implants. It depends, because when some people take a particular one; they begin to experience its side effects to the extent that some grow fatter. Lol … like me I use pills. P2: Lol … I use injectables. P3: Lol … am not using any, due to its side effects. P4: Lol … lol … I use the natural one, withdrawal, because it is more effective than the modern ones. FGD 5: Lol … I use the natural ones. P6: Lol … I use natural method. P7: Lol… it is the male condom we use.

***Male Group***- **P**1: I prefer abstinence, because of my religion. P2: Am using abstinence as well. P3: The natural ones - withdrawal. P5: The ones they have mentioned –abstinence and withdrawal. P6: I don't think is advisable for Christians using condom or any method for the wife. P7: What about abstinence? P6: Abstinence is different from using condom. Is it not a sin for a Christian to use condom? I think it's a sin to use modern method. P1: Is it not a sin that you use contraceptive? (**Igala Ethnic group**).

***Female Group*** - P1: I have used Injectables before. Now I am using pills. P2: I have used injectables, pills and male condom. P3: Using of pills, condom. P4: Am using injectables. P5: I use male condom, pills and sometimes herbs. P6: I use condom and herbs sometimes. P1: I use pills, because it is very effective for me.

***Male Group*** - P1: I have not used any method of family planning before. P3: I have not used it because am still young in marriage. P4: I only use condom **(Okun ethnic group)**.

The decision of whether or not a woman takes a modern contraceptive method was determined from the opinions of key informant interviewees. It was emphasized that the man plays a significant role in the decision to use a modern contraceptive method, and if a spouse opposes it, a woman is unable to use it. When asked who makes the decision to use a modern contraceptive method, these participants said:

It is the man that undertakes the decision on the use of contraceptive. If a man does not approve of it and a woman starts using it, in our culture, it means so many things. It could mean that such a woman is promiscuous or she has a hidden agenda. There are cases that when a woman used family planning without the approval of the husband in Ebira land that has led to serious issues in the family. Therefore, if a man does not approve it, no woman should try it **(KII/Ebira ethnic group)**.

It is the husband that must give approval. Although a woman can initiate the use of family planning, but if the husband says she should not use it, she doesn't have option. It is the husband. The husband has final decision on the use of any method whether traditional or modern (**KII/Igala ethnic group**).

It depends. Although women initiates the use of it, while the men gives approval in the past. But now a days, it is used to be a joint decision (KII/Okun ethnic group).

From the above narratives, the use of any form of contraception among women of reproductive age, especially modern ones, is predicated on the approval of their husbands. This shows that the influence of patriarchy on contraceptive use decisions affects women's decision to use contraception to avoid unwanted pregnancies.

Table 3 shows the adjusted and unadjusted odds ratio of modern contraceptive use for the selected minority ethnic groups within the same state in the North Central region of Nigeria. Unadjusted variables such as age, education, occupation, and family type were not significant to the use of modern contraceptives. In contrast, ethnicity, residence, religion, income and marital status were significant predictors of modern contraceptive use among minority ethnic when they were not adjusted.

**Table 3:** Results of binary logistic regression models on women currently using modern contraceptive by ethnic groups and background characteristics.

**Variables**	**Unadj. OR [95% CI for Exp(B)]**	**Adj. OR [95% CI for Exp(B)]**
***Ethnic group***		
Ebira (**ref**)	**1.000**	**1.000**
Igala	0.914 [0.650–1.284]	0.879 [0.611–1.266]
Okun	5.618 [4.068–7.759]^**^	4.803 [3.314–6.963]^**^
***Age***		
20–24	**1.000**	**1.000**
25–29	0.714 [0.342–1.490]	1.034 [0.443–2.417]
30–34	0.926 [0.452–1.899]	1.261 [0.548– 2.417]
35–39	0.949 [0.459–1.963]	1.205 [0.517–2.806]
40–44	1.252 [0.585–2.681]	1.507 [0.621–3.659]
45–49	0.881 [0.386–2.011]	0.775 [0.300–2.006]
***Residence***		
Rural (**ref**)	**1.000**	**1.000**
Urban	0.722 [0.588–0.935]^*^	0.642 [0.570–1.239]
**Education**		
No formal education	**1.000**	**1.000**
Primary	0.676 [0.383–1.190]	0.642 [0.332–1.239]
Secondary and higher	0.860 [0.601–1.198]	0.699 [0.477–1.029]
***Religion***		
Catholic (**ref**)	**1.000**	**1.000**
Other Christians	1.565 [1.018–2.405]^*^	1.412 [0.881–2.263]
Islam	0.499 [0.313–0.797]^*^	0.811 [0.476–1.381]
Traditionalists	0.744 [0.266–2.084]	0.894 [0.255–3.133]
***Occupation***		
Government employees	**1.000**	**1.000**
Private/self employed	0.958 [0.745–1.232]	1.245 [0.913–1.698]
Unemployed	1.297 [0.706–2.380]	1.587 [0.782–3.218]
***Average income per month***		
Below N30000 (**ref**)	**1.000**	**1.000**
N30000-N49999	1.434 [0.946–2.173]	1.608 [1.002–2.579]^*^
N50000 and above	1.431 [1.022–2.004]^*^	1.262 [0.843–1.890]
**Marital Status**		
Ever-married	**1.000**	**1.000**
Cohabiting	1.807 [1.048–3.116]^*^	1.816 [0.980–3.368]
Divorced/separated	1.235 [0.636–2.396]	1.532 [0.711–3.301]
***Family type***		
Monogamy	**1.000**	**1.000**
Polygyny	0.857 [0.631–1.165]	1.129 [0.777–1.642]

Significant at P < 0.05^*^ or 0.01^**^. The bold values represent the reference category (Ref. = 1.000) of each predictor variable.

The odds of using a modern contraceptive is significantly higher among the Okun women (UOR = 5.618, 95% CI 4.068–7.759) than the Ebira and Igala. There is no significant difference between the Ebira and Igala minority ethnic groups on modern contraceptive use. Urban residents were 27.8% less likely to use a modern contraceptive method than rural residents. The reasons given for as discussed among the participants in the focus group discussion were their preferences for traditional methods to modern due to some religious prohibitions and the fear of side effects. Higher monthly income is associated with higher chances of using modern contraceptives. Non-catholic Christians (UOR = 1.565, 95% CI 1.018–2.405) have higher use of a modern contraceptive method than Catholics. In contrast, Islam adherents have significantly lower odds (UOR = 0.499, 95% CI 0.313–0.797) than the Catholics. Furthermore, marital status is a strong predictor of modern contraceptive use among minority ethnic groups. Those who were cohabiting have higher odds (UOR = 1.807, 95% CI 1.048–3.116) than those who were ever married.

The predictors of modern contraceptive use among the minority ethnic groups changed slightly when all the variables were adjusted in the logistic regression; ethnicity and income remain the only significant variables. Although the odds were slightly reduced, Okun women still have the highest likelihood of using modern contraceptives than Ebira and Igala. The result shows no significant difference in the odds of modern contraceptive use between Ebira and Igala women of reproductive age. Participants who earned between N30000 and N49999 (AOR = 1.608, 95% CI 1.002–2.579) were 2 times more likely to use a modern contraceptive method than those who earned below the minimum wage of N30,000.

This study also investigated access to modern contraceptive methods through availability (locations of purchase) and pricing of current contraceptive methods to identify women's access to modern contraceptive methods. It was found that modern contraceptive methods can be obtained at reasonable prices from general hospitals (secondary facilities), clinics (Primary Health Centers) and pharmacies. As these participants narrated:

When we go to health centers for immunization, they use to give information about family planning methods. The natural ones we know were usually sourced from our grandparents. When you asked about how much they sell the modern types of contraception, I don't know that one but I know that the natural ones are not expensive which most of us are using with few using modern ones though still affordable (**Female FGD/Ebira ethnic group**).

… modern contraceptive is everywhere, they can be found in the general hospital, clinics and pharmacy with token. Some may even be free **(Female FGD/Igala ethnic group)**.

Most of the family planning methods are found in the clinics, general hospitals, pharmacy which ranged from N100, N200, N300 to N500 for condom and injectables methods. They are not too expensive. Even the natural ones too are even cheaper. Sometimes you get free ones. They are affordable and available everywhere. … the distance to get them are short. You may not pay more than N50 bike, if not a walking distance **(Female FGD/Okun ethnic group)**.

Given the above submissions from the three ethnic groups, it can be deduced that the locations where women can obtain modern contraceptive methods are not only close by, but also sold at reasonable prices, with some given to those who obtained them for free. This means that women's decisions about whether or not to utilize modern contraceptive methods are influenced by their ideological differences, not by the distance or cost of the methods. In other words, the use of modern contraceptive methods is mostly influenced by cultural orientations embedded in ethnicity, not by its availability.

## Discussion

The emphasis of this study was on differences in modern contraceptive use among three selected minority ethnic groups in North Central Nigeria: the Ebira, Igala and Okun. The Ebira and Igala ethnic groups, which are known for their high fertility, have a low use of modern contraceptives. The high prevalence of modern contraception among Okun women of reproductive age, on the other hand, is consistent with their low fertility, which may be traced back to Yoruba ethnic group in Southwest Nigeria, suggesting that modern contraceptive use has a cascading effect from this majority ethnic group (14, 32–34). This is similar to earlier research that has found ethnicity to be a strong predictor of contraceptive use (15, 29, 30).

Studies have shown that ethnicity as a factor is not a stand-alone predictor of contraceptive use on its own (6, 18, 24, 38, 39). Despite the fact that our study focuses on differences in contraceptive use across minority ethnic groups, these reproductive-age women live in both urban and rural areas, which has consequences for their reproductive health behavior. The residence, however, was found to be a significant predictor of modern contraceptive use as stand-alone variable. Women in urban centers were less likely than those in rural areas to use modern contraception. This contradicts earlier research that suggests women in urban regions are more likely to use modern contraception than women in rural areas (18, 21). This result could be due to the subtle influence of attitudes, values, and beliefs on contraceptive use, despite the fact that the majority of them live in cities. Perceived reasons identified in the qualitative study include the fear of its side effects, husband disapproval because of depletion of sexual pleasure and sin against religious beliefs. Another issue was the preference for traditional methods and their perceived efficacy, which discouraged most couples from using modern contraceptive methods in Ebira and Igala ethnic groups.

Religious membership has been linked to the use of modern contraceptives in other studies, as it impacts people's attitudes regarding reproductive health (14, 40). In this study, the likelihood of adopting a modern contraceptive method was higher among other Christians than Catholics. Those who practiced Islam, on the other hand, were less likely than those who practiced Catholicism to use a modern method of contraception. This supports (2), who discovered a higher propensity for modern contraceptive usage among Christians in Nigeria than among Muslims; and (27) who discovered non-use of modern contraceptives among various ethnic groups in Guinea due to religious prohibitions of one kind or another.

Previous studies have found a link between household income and the usage of a modern contraceptive method (4). The usage of modern contraception is also favorably connected with the respondents' economic level in this study. This suggests that if a woman's income rises, her likelihood of using a more effective contraceptive method rises as well. Other studies have also shown that a woman's marital status is a strong predictor of contraceptive use among ethnic groups (38, 39). Cohabiting women had a higher chance of utilizing modern contraceptive methods than married women. This suggests that, even among minority ethnic groups, cohabiting women, unlike married women, did not want or desire to limit the number of children they had. This result supports the findings of previous study (39) study that found differences in contraceptive use between married and cohabiting women.

The study has some limitations, despite the careful conduct of the research among the sample population. First, the study relied on self-reported data from participants, which may undermine the objectivity of the findings because responses from the subjects are rarely independently validated. Therefore, they could be vulnerable to a number of recollection biases that the researchers are unaware.

## Conclusion

In the context of this study, the current use of modern contraceptive methods among women in the selected minority ethnic is shaped, not only by ethnic identity, but also by specific socio-demographic factors of respondents. This has implications for achieving the Sustainable Development Goal 2030's objective 3.7. We, therefore, propose implementing reproductive health intervention programs aimed at educating minority ethnic groups about the benefits of modern contraception while also addressing individual contraceptive needs in Nigeria.

## Data Availability Statement

The raw data supporting the conclusions of this article will be made available by the authors, without undue reservation.

## Ethics Statement

The studies involving human participants were reviewed and approved by Kogi State Ministry of Health Ethical Review Committee, and University of Ibadan Social Sciences and Humanities Research Ethics Committee. The patients/participants provided their written informed consent to participate in this study.

## Author Contributions

AA and OF conceptualized the research topic. AA coordinated the field work and wrote the literature review, methodology, and analysis. OF reviewed and edited the paper. All authors contributed to the article and approved the submitted version.

## Conflict of Interest

The authors declare that the research was conducted in the absence of any commercial or financial relationships that could be construed as a potential conflict of interest.

## Publisher's Note

All claims expressed in this article are solely those of the authors and do not necessarily represent those of their affiliated organizations, or those of the publisher, the editors and the reviewers. Any product that may be evaluated in this article, or claim that may be made by its manufacturer, is not guaranteed or endorsed by the publisher.
